# New developments in the BI-RADS for MRI

**DOI:** 10.1590/0100-3984.2025.58.e2

**Published:** 2025-09-01

**Authors:** Tatiane Mendes Gonçalves de Oliveira

**Affiliations:** 1 Attending Physician at the Hospital das Clínicas da Faculdade de Medicina de Ribeirão Preto da Universidade de São Paulo (HCFMRP-USP), Radiologist at the Clínica Radiologia Especializada, Ribeirão Preto, SP, Brazil. Email: tatim@ hcrp.usp.br.

The role of subdivision of category 4 in the American College of Radiology (ACR) Breast Imaging Reporting and Data System (BI-RADS), with a malignancy risk ranging from 2% to 95%, is well-established in mammography and ultrasound. Subdividing BI-RADS category 4 allows more precise risk stratification of breast lesions that are suspicious for malignancy, promoting better understanding and multidisciplinary communication, as well as facilitating the assessment of the radiological-histopathological correlation after biopsy and contributing to quality control audits^(1)^.

Although not formally defined for use in breast magnetic resonance imaging (MRI) in the ACR BI-RADS, the benefits of subdividing category 4 could be even more significant for MRI. Given the high cost and limited availability of MRI-guided biopsy, enhancing risk stratification in category 4 might be crucial, especially for suspicious imaging changes on MRI that are not identified on mammography or ultrasound. For lesions with low malignancy risk, defined within category 4a, clinical follow-up could be considered as an alternative to MRI-guided biopsy.

In the article “Are we ready to stratify BI-RADS 4 lesions observed on magnetic resonance imaging? A real-world non-inferiority/equivalence analysis”, published in **Radiologia Brasileira,** Maltez de Almeida et al.^(2)^, unlike the authors of previous retrospective studies of the topic, presented results of a classification conducted during routine hospital practice, translating to practical applicability. Radiologists participating in the study had access to previous examinations and clinical data, allowing them to incorporate, albeit subjectively, mammographic and ultrasonographic findings into their decisions, which could have facilitated the stratification.

The Maltez de Almeida et al.^(2)^ study encompassed screening and diagnostic breast MRI examinations, with a total of 419 suspicious breast lesions, classified as 4a, 4b, or 4c according to ACR BI-RADS descriptors, which were divided into minor, intermediate, and major findings, achieving positive predictive values of 14.2%, 41.2%, and 77.2%, respectively, with statistical equivalence/noninferiority only for categories 4b and 4c.

Despite the relevance of the topic, there is a scarcity of studies on the stratification of BI-RADS category 4, including retrospective studies and studies that are methodologically heterogeneous. The image characteristics evaluated in the definition of subcategories, the number of descriptors included, the ratio between mass and non-mass lesions, and the ratio between benign and malignant lesions are factors with significant variability across studies^(1, 3, 4, 5, 6, 7, 8)^.

In general, the use of one or multiple image characteristics, such as spiculated margins, mass rim enhancement, segmental non-mass enhancement, and a clumped pattern or clustered ring pattern, appears to be effective in dividing breast lesions into categories 4b and 4c. However, the mere absence of these imaging findings that are highly specific for malignancy is not sufficient to define category 4a, for which the PPV shows greater variability (1.8–15.0%) in the literature^(6)^. These data illustrate how challenging it can be to establish a set of criteria capable of predicting a malignancy risk < 10% for patients undergoing breast MRI, who commonly present some additional risk factor for breast cancer and therefore have a higher pre-test probability of the disease.

Studies with extensive sampling and evaluation of additional parameters, such as T2 signal intensity and diffusion with apparent diffusion coefficient values, could help refine the criteria for subdividing BI-RADS category 4 for MRI^(4, 5, 9)^. Ultrafast sequences and their quantitative parameters (e.g., maximum slope, initial enhancement rate, and time between arterial and venous enhancement) have been shown to increase specificity in breast MRI and could eventually play a complementary role^(10, 11, 12)^. The use of radiomics and deep learning in breast MRI applied to contrast-enhanced sequences and diffusion has also produced promising initial results in differentiating between benign and malignant lesions and in stratifying the risk of suspicious findings^(13, 14)^. In addition, correlation with other methods such as mammography and ultrasound could help increase or decrease the positive predictive value of lesions identified on breast MRI, thus facilitating their upgrading or downgrading^(15)^.
